# Extracellular Vesicle Glial Fibrillary Acidic Protein as a Circulating Biomarker of Traumatic Brain Injury Severity

**DOI:** 10.1007/s12031-025-02360-5

**Published:** 2025-05-23

**Authors:** Ayad Babaee, Thea Overgaard Wichmann, Mikkel M. Rasmussen, Ole Brink, Dorte Aalund Olsen, Lars C. Borris, Maj Lesbo, Rikke Wehner Rasmussen, Carlos Salomon, Aase Handberg, Maiken Mellergaard, Claus V. B. Hviid

**Affiliations:** 1 Department of Clinical Medicine, Aarhus University, Aarhus, Denmark; 2 Department of Clinical Biochemistry, Aarhus University Hospital, Aarhus, Denmark; 3 Department of Neurosurgery, Aarhus University Hospital, Aarhus, Denmark; 4 Department of Orthopedic Surgery, Aarhus University Hospital, Aarhus, Denmark; 5 Department of Biochemistry and Immunology, Lillebaelt Hospital, University Hospital of Southern Denmark, Vejle, Denmark; 6 Department of Orthopedic Surgery, Regional Hospital Viborg, Viborg, Denmark; 7 Department of Clinical Biochemistry, Aalborg University Hospital, Aalborg, Denmark; 8 Department of Clinical Medicine, Aalborg University, Aalborg, Denmark; 9 Department of Surgery and Intensive Care, Regional Hospital Viborg, Viborg, Denmark; 10 Translational Extracellular Vesicles in Obstetrics and Gynae-Oncology Group, Faculty of Medicine, University of Queensland Centre for Clinical Research, Royal Brisbane and Womens Hospital, The University of Queensland, Brisbane, QLD 4029 Australia; 11 UQ Centre for Extracellular Vesicle Nanomedicine, The University of Queensland, Brisbane, 4029 Australia

**Keywords:** Extracellular vesicle, Glial fibrillary acidic protein, Traumatic brain injury, Biomarkers

## Abstract

Traumatic brain injury (TBI) remains a major global health challenge with a need for improved diagnostic and prognostic biomarkers. This study aimed to evaluate the biomarker potential of extracellular vesicle (EV)-encapsulated glial fibrillary acidic protein (EV-GFAP), neurofilament light chain (EV-NfL), total tau (EV-T-Tau), and ubiquitin carboxy-terminal hydrolase L1 (EV-UCH-L1) in TBI. A cohort of 93 trauma patients (75 with TBI and 18 without TBI) was analyzed. Patients were sampled on admission, as well as 15 and 72 h post-injury. Following initial method validation, EVs were isolated from plasma using size exclusion chromatography (SEC), and plasma levels and EV cargo levels of biomarkers were measured using an ultra-sensitive Single Molecule Array. EV-GFAP levels were significantly elevated in TBI patients compared to non-TBI trauma patients at admission and 15 h. A positive head CT was associated with 2.85 (95% CI: 1.18–6.91) fold increased EV-GFAP, whereas EV-NfL, EV-T-Tau, and EV-UCH-L1 levels were not affected. None of the tested EV biomarkers were associated with 1-year mortality or 6–12 months’ functional outcome. Plasma-GFAP levels increased 3.4 (95% CI: 1.72–6.70) fold with a positive head CT but were not associated with outcomes. EV-GFAP shows potential as an early biomarker of TBI, but plasma-GFAP remains a practical and reliable alternative. Future studies should explore the potential complementary roles of EV-based biomarkers on alternative aspects of TBI pathophysiology and prediction of long-term outcomes. Studies should refine methods to enhance reproducibility and clinical applicability.

**Supplementary Information:**

The online version contains supplementary material available at 10.1007/s12031-025-02360-5.

## Background

Traumatic brain injury (TBI) is a significant global health concern, affecting approximately 69 million people annually (Dewan et al. 2018). TBI is a complex condition covering a wide range of severities with an equally wide range of long-term outcomes posing a clinical challenge and calls for improved diagnostic and prognostic approaches. Enhancing our ability to predict long-term outcomes and identify patients who may benefit from specialized interventions or rehabilitation has considerable potential in terms of personal and economic consequences.

Several studies have investigated freely circulating, nerve-derived proteins such as glial fibrillary acidic protein (GFAP), neurofilament light chain (NfL), ubiquitin carboxy-terminal hydrolase -L1 (UCH-L1), and total Tau (T-Tau) as biomarkers of TBI (Papa et al. 2016; Lei et al. 2015; Abdelhak et al. 2022; Helmrich et al. 2022). These biomarkers have shown strong and cumulative diagnostic and prognostic utilities across the TBI severity spectrum (Helmrich et al 2022; Bazarian et al. 2018; Kaaber et al. 2024).

Besides circulating freely in plasma, nerve-derived proteins are actively shed from nerve-tissue within extracellular vesicles (EVs) (Nekludov et al. 2017). EVs are lipid bilayer particles secreted by all cell types that encapsulate proteins, nucleic acids, and lipids from the cells of origin. Hereby, they provide a circulating, liquid biopsy of the originating cells, which may reflect the biological state of the host tissue more precisely than freely circulating proteins (Abels and Breakefield 2016; Khan et al. 2022). As EVs readily cross the blood–brain barrier (BBB), they may constitute an easily available, stable, and non-invasive source of biomarkers that accurately reflect the status of nerve cells in TBI patients.

Despite this, only few studies have investigated the biomarker potential of EV cargo proteins during the initial phases of acute TBI (Mondello, et al. 2020; Kawata et al. 2018; Guedes et al. 2022; Goetzl et al. 2019). Three of these studies were small (Mondello, et al. 2020; Kawata et al. 2018; Goetzl et al. 2019) and only one study provided longitudinal data (Mondello, et al. 2020). Furthermore, the majority of evidence is based on precipitation-based methods for EV isolation (Mondello, et al. 2020; Guedes et al. 2022; Goetzl et al. 2019) and lack thorough methodological characterization, which may raise concerns about the purity and quality of the isolated EVs (Brennan et al. 2020). This underscores the need for additional research, using optimized and validated isolation and characterization protocols for EVs and down-stream analytical methods, to determine if EVs provide a superior source of biomarkers in acute TBI.

In this study, we address the limitations of prior reports by providing a thoroughly validated method for EV-cargo analysis in TBI patients. We apply this method on a large acute TBI cohort to analyze acute and longitudinal changes in the nerve-derived proteins GFAP, NfL, UCH-L1, and T-Tau. Lastly, we compare the biomarker abilities of these EV-cargo proteins with those of their counterparts freely circulating in plasma.

## Materials and Methods

### Study Population

This study was carried out using biobank samples from the established Systematic Urine Evaluation for Activation of Coagulation in Severe Trauma (SURVIVE) cohort (Kaaber et al. 2024; Lesbo et al. 2023). The cohort comprises 418 trauma patients admitted to the level I trauma center at Aarhus University Hospital between March 2017 and March 2018. All admitted trauma patients were assessed for eligibility in the SURVIVE study. The following exclusion criteria were applied: (1) individuals younger than 18 years; (2) patients who were dead upon arrival; (3) declared pregnancy; (4) those who did not meet Advanced Trauma Life Support (ATLS) criteria for trauma team activation; and (5) individuals who opted to decline or withdraw consent.

For this study, a nested case–control cohort was established through a two step-process. Initially, fifty trauma patients with a positive head CT (defined as any acute intracranial pathology visible on CT) and a Marshall Classification Score (Marshall score) > 1 was extracted from the database and selected as cases (see Data Collection for description). These were matched by age, sex, and Injury Severity Score (ISS) with 50 controls, who were trauma patients with a negative head CT and a Marshall score of 1. To ensure the control group was free of head trauma, a second classification of the control group was performed by a detailed chart review. In this step, control patients with any documentation of head injury in the chart were reclassified as cases and cases with concomitant traumatic spinal cord injury (*n* = 7) were excluded, resulting in a total of 93 individuals for analysis.

### Data Collection

In the SURVIVE study, patient data was collected from the Aarhus University Hospital trauma registry and the electronic medical records. The trauma registry provided detailed information on the time of injury, injury mechanism, prehospital treatment interventions, vital signs (heart rate, respiratory rate, and blood pressure), Glasgow Coma Scale (GCS), and Abbreviated Injury Scale (AIS) score at admission. The AIS score was used to calculate the New Injury Severity Score (NISS). For patients with missing GCS data in the registry, values were retrieved from admission records.

The electronic medical records provided information on patient demographics, including age and sex, as well as clinical data, including time of hospital admission, blood sampling, antiplatelet or anticoagulant therapy, surgical procedures, and blood transfusions within 72 h of admission. If antiplatelet or anticoagulant therapy status was missing, patients were classified as not receiving this treatment.

The initial head CT scans were reviewed to assign Marshall CT Classification scores. The Marshall score, a six-category system based on midline shift, basal cistern compression, and hemorrhagic lesions, was recorded. Patients with head trauma, but no visible intracranial pathology were assigned a score of 1 (Marshall et al. 1992). CT scans were evaluated by a junior doctor with neurosurgery experience and verified by a senior consultant in neurosurgery. Outcome measures included 1-year all-cause mortality and functional outcome assessed by the Glasgow Outcome Scale Extended (GOSE) at 6–12 months (Wilson et al. 1998). Functional outcomes were classified as favorable (GOSE ≥ 5) or unfavorable (GOSE ≤ 4), based on medical records documenting patient consciousness, independence, and social or work engagement.

### Blood Samples

Blood samples were collected upon arrival to the trauma resuscitation room and again at 15 (± 3) hours and 72 (± 6) hours after admission. If patients were discharged or transferred to another hospital within 72 h, they exited the study and further blood sampling was discontinued.

Blood samples were obtained either from an arterial line or by venous puncture, and collected into citrate, heparin and EDTA tubes (BD Vacutainer®, Becton, Dickinson and Company, Franklin Lakes, NJ, USA). Samples were transported to the laboratory at room temperature (RT) and processed within 1 h. For chemistry and hematology analyses, all procedures followed the standard operating procedures established in our accredited clinical laboratory (DS/EN ISO15189). Chemistry samples (heparin tubes) were centrifuged at 3000* g* for 5 min at 22–24 °C before analysis. Hematology samples (EDTA tubes) were analyzed using whole blood. Samples intended for biomarker analysis (citrate tubes) were centrifuged at 3000* g* for 25 min at 20 °C, then frozen at − 80 °C pending biochemical analysis.

For the method validation studies, citrate anti-coagulated blood samples from nine randomly selected volunteers were collected. The samples were anonymized and pooled into three pools (A, B, C), with each pool containing blood from three different individuals, and stored at − 80 °C until analysis. The study to assess the collective reproducibility of the established EV isolation combined with analysis of nerve-derived proteins in the EV cargo was performed using a citrate plasma pool from 10 random volunteers. The 10 plasma samples were mixed, and aliquoted into 500-μL tubes. One aliquot was used for EV isolation on ten consecutive days and the isolates were stored at − 80 °C until being batch analyzed by Simoa.

### Biochemical Analysis

Chemistry parameters (such as creatinine) and hematology parameters (such as hematocrit) were measured at the Department of Clinical Biochemistry at Aarhus University Hospital using validated assays for standard clinical practice. The Roche Cobas 6000 system was utilized for chemistry analyses, while the Sysmex XE-5000 was employed for hematology measurements.

The Size Exclusion Chromatography (SEC)-enriched samples were analyzed at the Department of Biochemistry and Immunology, Lillebaelt Hospital, between June 2024 and July 2024. The analysis was done using the Neurology 4-plex assay B kit (Quanterix Corp, MA, USA) on the ultra-sensitive Single Molecule Array (Simoa) HD-X Analyzer (Quanterix Corp) according to the manufacturer’s instructions. A certified laboratory technician, experienced in Simoa techniques and blinded to the study groups, performed all analyses. For these analyses, citrated plasma and EV lysate were thawed at RT and analyzed in batches. Citrated plasma was subjected to a standard four-fold dilution. Assay performance was monitored using three quality controls per run: two manufacturer-provided and one in-house plasma pool. Detailed information on the analytical performance is provided in Supplementary Table S1 (for details on the number of samples below the limit of detection (LOD), blank, or missing across the three time points for EVs and plasma, see Supplementary Table S2).

### Extracellular Vesicle Isolation

Citrated plasma was thawed at RT and centrifuged at 1500 *g* for 10 min at 4 °C, to remove large debris. The supernatant was carefully transferred to a new tube and centrifuged again at 10,000 *g* for 10 min at 4 °C to further clarify the sample.

Commercially available 70-nm qEVoriginal size exclusion columns (ICO-70, IZON, Christchurch, New Zealand) were used according to the manufacturer’s instructions. Five hundred microliters of the citrated plasma was loaded onto the column and eluted with phosphate-buffered saline (PBS) (Merck, D8537). An Automatic Fraction Collector (IZON, Christchurch, New Zealand) was employed to automate the column runs. A volume of 1.5 mL was collected after an initial buffer volume of 2.7 mL was eluted. Each column was used up to ten times, as specified by the manufacturer. Flushing steps were performed between samples to prevent carryover contamination. 8.5 mL of 0.5 M NaOH (Merck, 1.06498.1000) was used to wash the column, followed by another 17 mL PBS flush to remove any residual NaOH. Before the first use of each column, it was flushed with 17 mL of PBS to equilibrate the column. The collected 1.5-mL SEC-enriched samples were pipette-mixed to ensure homogeneity, then aliquoted into three 500-μL tubes and stored at − 80 °C until further analysis. To minimize the risk of EV degradation, all samples underwent only a single freeze/thaw cycle.

### Nanoparticle Tracking Analysis

Particle size, concentration, and distribution were analyzed with the Nanoparticle Tracking Analysis (NTA) device (Zeta View PMX-420-Quatt, Particle Metrix GmbH, Germany) using light scatter mode (488 nm). Samples were prepared and diluted in PBS (Cat. no. D8537) to reach a concentration suitable for analysis (between 150 and 300 particles/frame). Prior to analysis, the instrument was calibrated using 100-nm polystyrene standard beads (Cat. no. 700074, Particle Metrix GmbH, Germany; 1:250.000 dilution in H_2_O). Measurements were performed from three exposures at 11 camera positions. Minimum brightness was set to 30 a.u., shutter to 100 a.u., the minimum area to 10 a.u., maximum area to 1000 a.u., tracking radius to 100 a.u., minimum track length to 15 a.u., and temperature to 23 °C. Particle size distributions were calculated using the ZetaView software (version 8.05.16).

### Nanodrop

Protein concentrations were measured using the NanoDrop One spectrophotometer (ThermoFisher Scientific, Wilmington, DE, USA) following the manufacturer’s guidelines. Briefly, 2 μL of each sample was loaded onto the measurement pedestal and absorbance was measured at 280 nm (A280). The instrument was calibrated with a blank PBS solution before each measurement.

### High-Resolution Flow Cytometry

SEC-enriched samples were thawed and stained with antibody mix solutions, prepared as previously described (Rasmussen, et al. 2021). Antibodies used for analysis were FITC-conjugated mouse monoclonal anti-CD9 (Biolegend, Cat. no. 312104), FITC-conjugated mouse monoclonal anti-CD81 (Biolegend, Cat. no. 349504), FITC-conjugated mouse monoclonal anti-CD63 (Biolegend, Cat. no. 353006), and matched isotype: FITC-conjugated mouse IgG1κ (Biolegend, Cat. no. 400110). Antibody and PBS (D8537, Merck) mixes were prepared directly on 0.45-μm centrifugation filters (UFC30HV25, Merck, Darmstadt, Germany) as described in Supplementary Table S6.

For staining, 90 μL of sample was transferred to a new tube followed by the addition of 50 μL of master or isotype solution. Samples were mixed thoroughly by pipetting and incubated for 2 h at RT in the dark. Next samples were diluted individually in PBS to obtain an equal flow rate (2000–4000 events/s) on the flow cytometer (determined by titration of the unlabeled sample). Diluted samples were kept dark at RT until analysis. Antibody aggregates in the antibody and isotype mix solutions were tested by analyzing the solutions in PBS. Lastly, detergent lysis control was included for all stained samples to verify the staining of EV populations. Here, a master mix-stained sample was incubated for 30 min at RT in the dark with 1% v/v Triton X-100 (Cat. no. 93443, Merck, Darmstadt, Germany) or stained samples were sonicated as described below. Samples were prepared for analysis in a flat-bottom 96-well plate (AH Diagnostics, Cat. no. 064026) by autosampler. Analysis was done using an Apogee A60Micro-PLUS high-resolution flow cytometer (Apogee Flow Systems, Hemel Hempstead, UK), as previously described (Bartko et al. 2024), specific setting in Supplementary Table S6 and S7. Data were recorded in the Histogram software (version 6.0.139, Apogee Flow).

Daily performance of the flow cytometer was ensured using Apogee Mix bead mixture (Cat. no. 1527, Hemel Hempstead, Hertfordshire, UK) to ensure the flowrate and light scatter signal stability and Rainbow Calibration particles (Cat. no. RCP-30–5, Spherotech, Lake Forest, Illinois, USA) to verify the signal stability in the fluorescence channels. Moreover, Rosetta Calibration Beads (Cat no. CAL003, Exometry, Amsterdam, The Netherlands) to convert arbitrary light scatter signals to size (in nm). Data were analyzed using the FlowJo software version 10 (TreeStar, Ashland, OR, USA). Gating was done as shown in Figure S1 and EV concentrations of CD9^+^ CD63^+^CD81^+^-positive EVs (EVs/mL) were calculated using the equation: EVs/mL = ((CD9^+^ CD63^+^CD81^+^-positive EVs)/(volume of the run sample)) * sample dilution factor * 1,000,000.

### EV Lysis

SEC-enriched samples intended for biomarker measurement were lysed using Triton X-100 (#93,443, Merck). The samples were thawed and mixed with 1% Triton X-100 (final concentration). The mixture was vortexed and incubated at RT for 30 min prior to biomarker measurement.

Sonication was done with a Bioruptor UCD-200 sonication device (Diagenode, Liège, Belgium). SEC-enriched samples were placed in a cooling bath to maintain a consistent temperature and prevent overheating during the sonication process. The sonication was performed in cycles. Sonication for 30 s (ON) followed by a 15s rest period (OFF) constituted one cycle. This process was repeated for a total of 6 cycles.

### Statistical Analysis

Data distribution was evaluated visually through inverse QQ-plots and statistically using the Shapiro–Wilk W test for normality. Data is presented as absolute numbers (*n*) with percentages, means with standard deviation (SD) or 95% confidence intervals (CI), and medians with interquartile range (IQR) when appropriate. For categorical data, we used the chi-squared test, while continuous variables were compared with the Mann–Whitney *U*-test. To adjust biomarker level comparisons between TBI patients and non-TBI trauma patients, we used multiple linear regression, setting biomarker levels as the dependent variable and TBI status as the independent variable, with adjustments for age, sex, and NISS.

Within the TBI group, we applied multiple linear regression to examine the impact of increasing TBI severity on biomarker levels. In each model biomarker levels were the dependent variable and admission GCS, presence of a lesion on head CT, and the Marshall Classification score as independent variables. We categorized admission GCS as mild (14–15), moderate (9–13), or severe (3–8). The presence of a head CT was dichotomized, and the Marshall Classification score was grouped into three categories (I, II–IV, V–VI). All models were adjusted for age, sex, and NISS. As biomarker levels displayed skewed distributions, they were ln-transformed before the regression analysis, with results then back-transformed to the original scale for interpretation. Each model was checked to ensure it met standard assumptions of multiple regression analysis. The association between biomarker levels, all-cause mortality and functional outcome was analyzed by logistic regression. A significant level of 5% was used for all analyses. All statistical analyses were performed using Stata/SE 18.0 for Windows. Figures were created using GraphPad Prism for Windows, Version 10.3.1.

## Results

### Method Validation

**Fig. 1:**
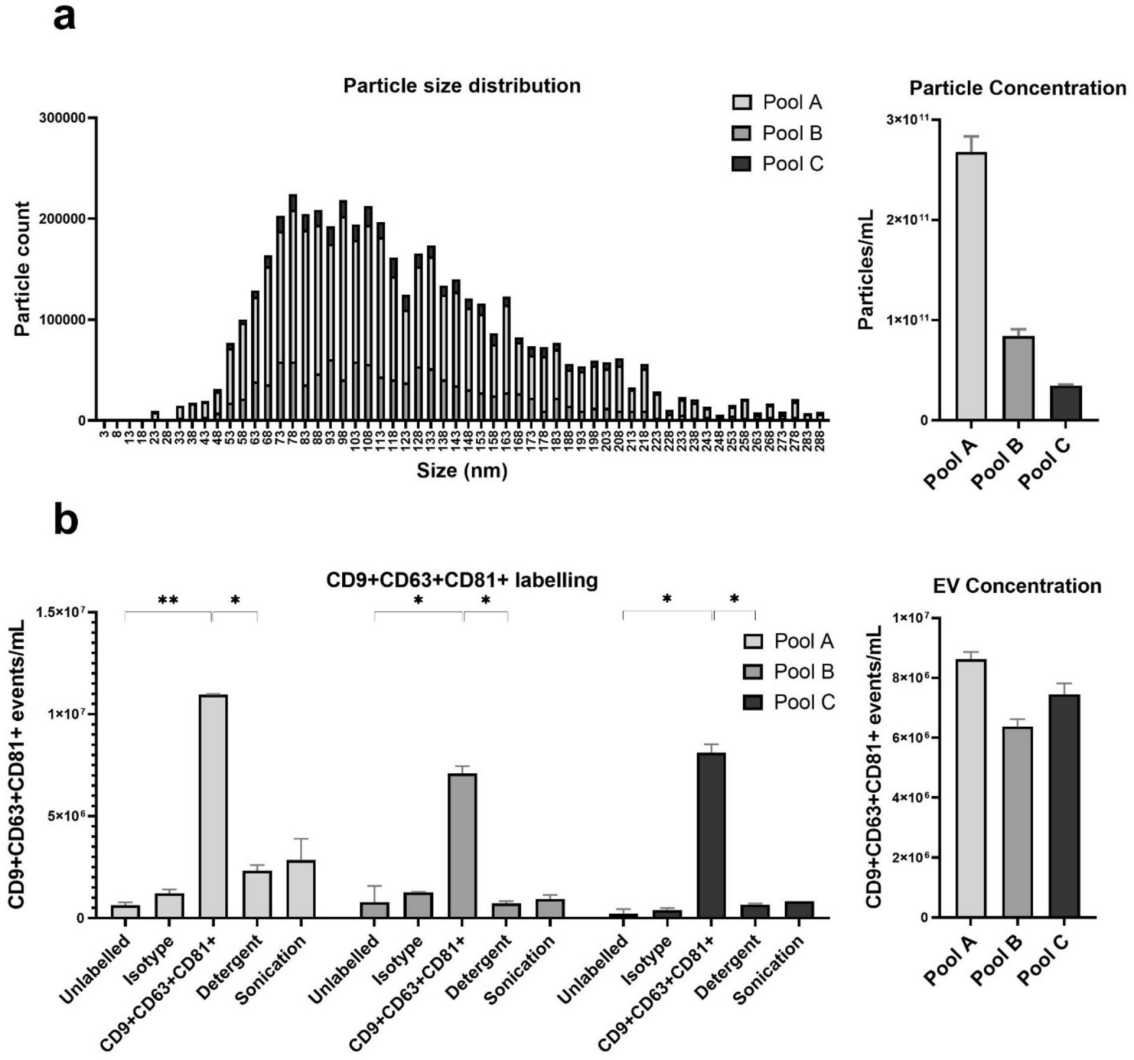

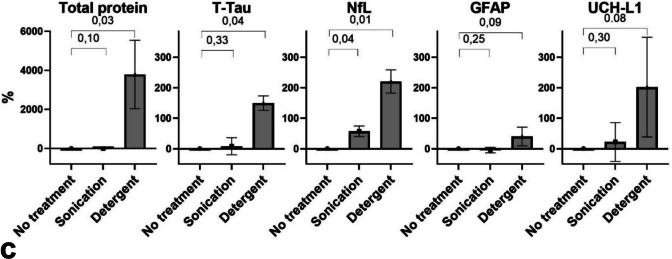
Validation of SEC purification and EV-cargo analysis. **a** Particle size distribution (left) and concentration (right) after SEC purification were evaluated by NTA in three donor pools (pool A, pool B, pool C). The size distribution of the purified EVs is shown as particle counts across size ranges, and the total particle concentration in the collected volume is presented as particles per milliliter. **b** Flow cytometry analysis of SEC-enriched samples from the same three donors (pool A, pool B, pool C). Results for CD9 + CD63 + CD81 + extracellular vesicle counts in the three pools are shown for unlabeled samples, after the addition of Isotype IgG or CD9 + CD63 + CD81 + IgG antibodies, or in samples labeled with CD9 + CD63 + CD81 + and treated with detergent (detergent treated) or sonicated. The right panel shows CD9 + CD63 + CD81 + positive counts after detergent subtraction. **c** Protein concentration in the SEC-enriched samples from the three donors, prior to and after detergent treatment or sonication. Total protein was measured using NanoDrop and the four nerve-specific biomarkers GFAP, NfL, T-Tau, and UCH-L1 were measured by single molecule array under the same conditions. Results are expressed as a percentage increase relative to the untreated baseline. The absolute concentrations measured (pg/mL) were nanodrop [no treatment: 1.30, sonication: 1.15, detergent: 42.52], Simoa: [GFAP, no treatment: 11.96, sonication: 11.41, detergent: 17.85, NfL, no treatment: 0.30, sonication: 0.45, detergent: 0.85, T-Tau, no treatment: 0.35, sonication: 0.40, detergent: 0.56, UCH-L1, no treatment: 19.6, sonication: 20.76, detergent: 47.00]. *p* < 0.05 (*), *p* < 0.01 (**). Abbreviations: GFAP, glial fibrillary acidic protein; NfL, neurofilament light chain; T-Tau, total Tau; and UCH-L1, ubiquitin carboxy-terminal hydrolase-L11

Based on the elution profiles (Supplementary Fig. S1), SEC-enriched samples were collected from fractions 7–10 (1.5 mL) after discarding the initial 2.7 mL to minimize protein contamination. The particle size distribution after SEC enrichment as evaluated by NTA analysis, is presented in Fig. 1a. The particle size distribution in the three donor pools fell mostly within the 70–130-nm range (left panel) and the number of particles per mL enriched in each donor pool as measured by NTA varied substantially (right panel).

High-resolution flow cytometry (hFCM) was used to validate the NTA findings. Labeling with CD9, CD63, and CD81 confirmed the presence and determined the concentration of EVs in the SEC-enriched sample from the three donor pools (Fig. 1b). Detergent treatment or sonication reduced the CD9^+^CD63^+^ CD81^+^ signal to the background control levels (isotype and unlabeled). The EV-particle count by hFCM was estimated by subtraction of the detergent signal from the CD9^+^CD63^+^CD81^+^ signal and showed only little variation between the donor pools, Fig. 1b, right panel.

To confirm the ability to measure EV protein cargo, the total protein content in the SEC-enriched sample from the three donor pools was measured before and after detergent treatment or sonication (Fig. 1c). Detergent treatment resulted in a substantial increase in protein content in the SEC-enriched sample, while sonication caused only a minimal change compared to the untreated condition. The changes of GFAP, NfL, T-Tau, and UCH-L1 in the SEC-enriched sample was also measured under these treatment conditions. Detergent treatment led to an increase in the levels of all four biomarkers, while sonication caused only minor changes relative to the untreated baseline.

Lastly, the collective reproducibility of the established method was evaluated. EVs from a plasma pool was SEC-enriched and treated with detergent. This was done ten times using the same plasma pool. The ten SEC-enriched samples were then batch analyzed for NfL, GFAP, T-Tau, and UCH-L1. The resulting CV% values were 38% for NfL, 36% for GFAP, 23% for T-Tau, and 23% for UCH-L1.

### EVs in TBI

Characteristics of TBI and non-TBI trauma patients are presented in Table 1. Of 93 patients included, 75 patients were classified as having TBI, while 18 patients were classified as non-TBI. The cohort was predominantly males and no significant difference in sex distribution or age was observed between TBI and non-TBI. A significantly larger proportion of TBI patients than non-TBI patients required intubation while no differences were observed for NISS score in the two groups. Likewise, there was no significant difference in the proportion of patients who received a blood transfusion within 72 h.

**Table 1:** Baseline characteristics of study cohort

Characteristics	TBI (*n* = 75)	Non-TBI (*n* = 18)	*p* value
Sex, males	64 (85)	14 (78)	0.43
Age, years	62 (38–73)	62 (56–67)	0.73
**Preadmission medical treatment and biochemistry**			
Anticoagulant prescription	8 (11)	1 (5)	0.33
Platelet inhibitor prescription	11 (14)	1 (5)	0.15
Hematocrit, Vol.frac RI: male: 0.40–0.50 RI: female: 0.35–0.46	0.41 (0.39–0.44)	0.41 (0.39–0.45)	0.67
Hemoglobin, mmol/L RI: male: 8.3–10.5 RI: female: 7.3–9.5	8.6 (8.2–9.3)	8.7 (8.2–9.2)	0.75
Creatinine, μmol/L RI: male: 60–105 RI: female: 45–90	83 (71–96)	84 (72–91)	0.90
C-reactive protein, mg/L RI: < 3.0	1.2 (0.6–2.7)	1.3 (0.9–4.1)	0.28
Leukocytes, × 10^6^/L RI: 3.5–10.0	11.0 (8.3–16.1)	10.0 (9.1–12.3)	0.35
Fibrinogen, μmol/L RI: 5.5–12.0	7.7 (6.5–9.0)	8.4 (7.0–9.5)	0.22
D-dimer, mg/L RI: < 0.5	9.5 (2.8–19.0)	8.7 (5.7–14.2)	0.86
**Clinical severity on admission**			
Intubated on admission,	24 (32)	1 (5)	0.02
Shock index, > 0.8	11 (15)	1 (5)	0.24
Glasgow Coma Scale (GCS)			
14–15	37 (49)	16 (88)	< 0.01
9–13	24 (32)	0 (0)	< 0.01
3–8	13 (17)	1 (5)	0.21
New Injury Severity Score (NISS)			
0–8	6 (8)	3 (16)	0.26
9–15	14 (18)	6 (33)	0.17
16–24	26 (34)	4 (22)	0.31
24	29 (38)	5 (28)	0.39
Type of injury			
Blunt	72 (96)	18 (100)	1.00
Penetrating	1 (1)	0 (0)	1.00
Mechanism of injury			
Fall	26 (36)	4 (22)	0.31
Traffic accidents	33 (44)	6 (33)	0.41
Violence or suicide	3 (4)	1(5)	0.77
Other	13 (17)	7(39)	0.05
Surgical procedure within 72 h	44 (58)	9 (50)	0.50
Blood transfusion within 72 h	13 (17)	2 (11)	0.51

All values are reported as *n* (%) or median with interquartile range (IQR). Abbreviations: *GCS* Glasgow coma scale, *NISS* new injury severity score, *RI* reference interval.

### Biomarker Levels in the EV Cargo

**Fig. 2:**
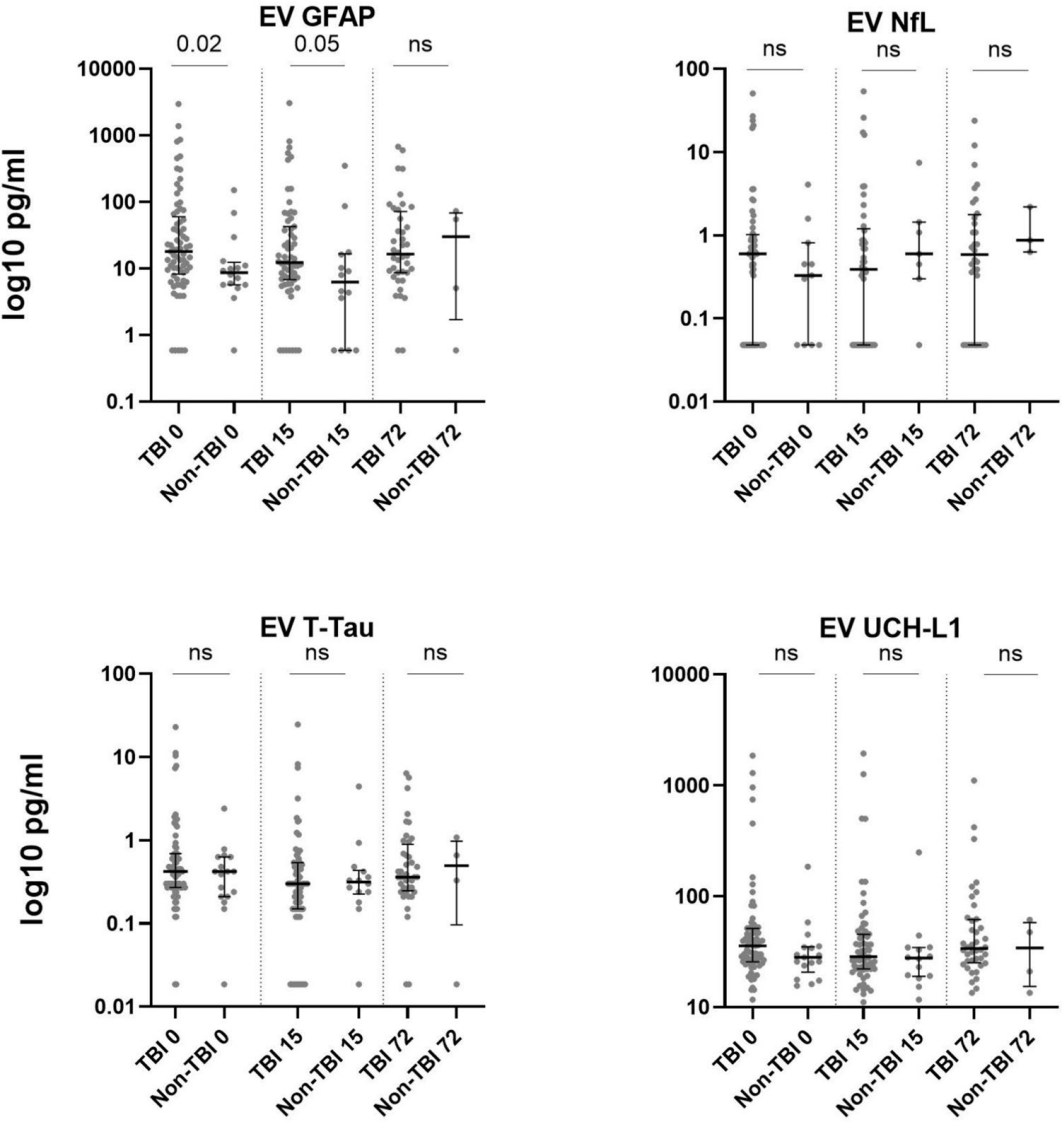
EV cargo protein in trauma patients with and without TBI. Biomarker concentration in extracellular vesicle cargo from patients with TBI and non-TBI on hospital admission (0 h), and after 15 and 72 h of hospital stay. Biomarker levels are presented on a logarithmic scale (log10 pg/mL). Values are presented as median with interquartile ranges (IQR). Abbreviations: TBI, traumatic brain injury, GFAP glial fibrillary acidic protein, NfL neurofilament light chain, T-Tau total Tau, UCH-L1 ubiquitin carboxy-terminal hydrolase-L1, ns not significant

The EV cargo levels of GFAP (EV-GFAP), NfL (EV-NfL), T-Tau (EV-Tau), and UCH-L1 (EV-UCH-L1) were measured at admission, 15 h, and 72 h after injury (Fig. 2). EV-GFAP levels were significantly higher in TBI patients than non-TBI patients at admission (*p* = 0.02) and 15 h (*p* = 0.05), but not at 72 h. To assess diagnostic performance, we performed a receiver operating characteristic (ROC) analysis for EV-GFAP. At admission EV-GFAP yielded an area under the curve (AUC) of 0.68 (95% CI: 0.54–0.82), indicating moderate discriminatory ability between TBI and non-TBI patients. EV-NfL, EV-Tau, and EV-UCH-L1 levels were not different at any of the investigated time points.

**Fig. 3:**
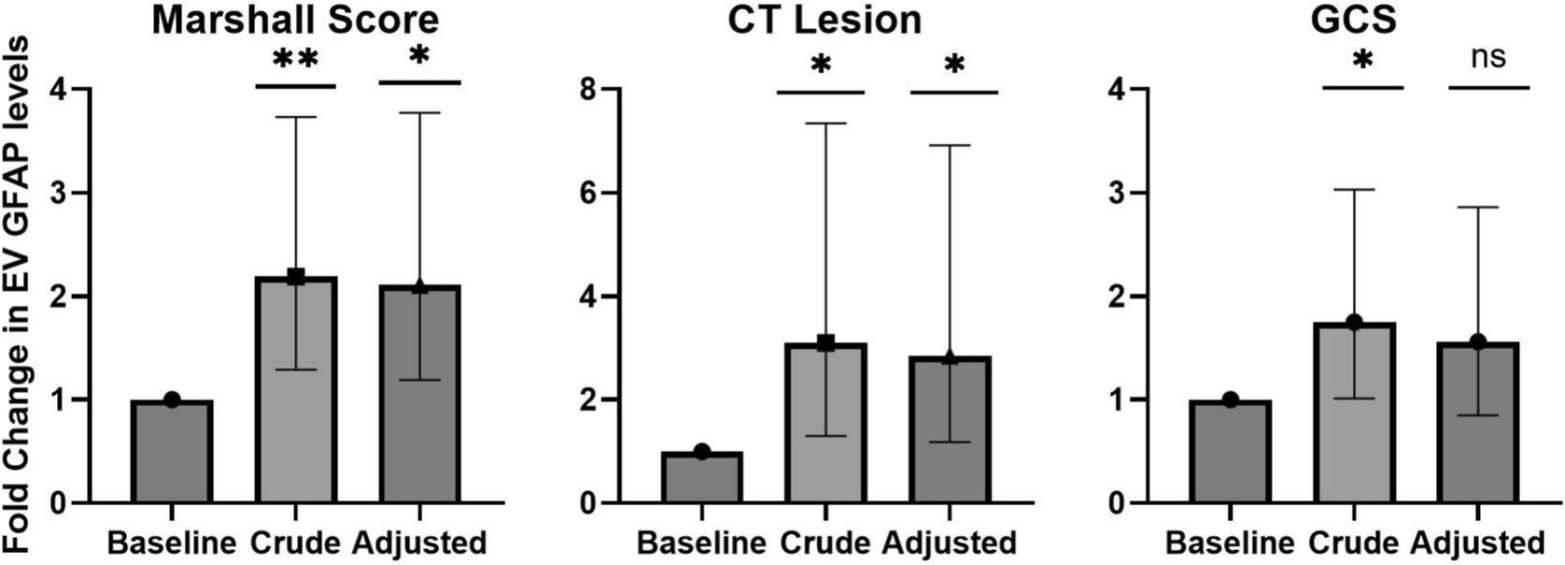
Association of EV-GFAP with TBI severity indicators. Association between extracellular vesicle GFAP cargo levels and TBI severity indicators at admission. The effect of increasing Marshall score (I, II–IV, V–VI), presence of a positive head CT (yes/no), and reduction in GCS (14–15, 9–13, 3–8) score is shown. Bars represent fold changes in EV GFAP cargo levels for crude models and after adjustment for age, sex, and NISS. For all three indicators, baseline represents the best clinical category. Error bars represent 95% confidence intervals. *p* < 0.05 (*), *p* < 0.01 (**), *p* ≥ 0.05 (ns) against baseline. Abbreviations: NISS new injury severity score, GCS Glasgow Coma Scale, CT computed tomography, GFAP glial fibrillary acidic protein, ns not significant

The associations between EV cargo biomarker levels and TBI severity indicators at admission were analyzed (Fig. 3). EV-GFAP levels were associated with increasing Marshall score. In the crude model, GFAP levels increased 2.19-fold (95% CI: 1.29–3.73, *p* < 0.01) with increasing Marshall score (I, II–IV, and V–VI). This association was unaffected by adjustment for age, sex, and NISS [2.11-fold increase (95% CI: 1.19–3.77, *p* = 0.01)]. The presence of a positive head CT was associated with 3.10 times (95% CI: 1.30–7.34, *p* = 0.01) higher EV-GFAP level in the crude model which remained significant [2.85-fold increase; (95% CI: 1.18–6.91, *p* = 0.02)] after adjustment for age, sex and NISS. In contrast, a reduced GCS score was associated with a 1.75-fold increase (95% CI: 1.01–3.03, *p* = 0.04) in EV-GFAP levels, but this association disappeared after the adjustment for age, sex, and NISS [1.56-fold reduction (95% CI: 0.86–2.86, *p* = 0.15)].

EV-Tau, EV-NfL, and EV-UCH-L1 levels at admission were not associated with TBI severity indicators in either the crude or adjusted models (*p* > 0.05 for all comparisons) (Supplementary Table S3). The association between admission EV cargo biomarker levels, 1-year mortality, and 6–12 months’ functional outcome among TBI patients was analyzed by logistic regression modeling (Supplementary Table S5). None of the biomarkers were associated with 1-year mortality or 6–12 months’ unfavorable outcome (*p* > 0.05).

### Plasma Biomarker Levels

Biomarker levels were also analyzed in citrated plasma (Supplementary Fig. 2). Plasma-GFAP levels were elevated in TBI patients compared to non-TBI trauma patients at all time points (all, *p* < 0.03). NfL and UCH-L1 levels were elevated at 15 h only (*p* < 0.01) while T-Tau plasma levels showed no significant differences.

The association between plasma biomarkers and TBI severity indicators uncovered significant and consistent associations with GFAP only (Supplementary Table S4). The magnitude of the adjusted associations was GCS: 0.61 (95%CI: 0.37–0.88), positive head CT: 3.4 (95% CI: 1.72–6.70), increasing Marshall score: 2.1 (95% CI: 1.33–3.31). None of the plasma biomarker levels were consistently associated with the investigated outcomes (Supplementary Table S6).

## Discussion

This study shows elevated EV-GFAP levels in trauma patients with TBI compared to trauma patients without TBI, and an association between EV-GFAP levels and the severity of TBI. These findings, obtained through rigorously validated methods for EV isolation and protein measurement, support a potential of EV-associated biomarkers in TBI.

EV research has methodological challenges related to contamination with non-EV particles, variability in yield, and purity of isolates (Witwer, et al. 2013). Among isolation methods, some methods prioritize high yield but compromise on purity, while others achieve greater purity at the expense of time efficiency, cost, or reproducibility (Allelein et al. 2021). Findings derived from EV research are therefore heavily dependent on the isolation method used.

In this study, SEC was used, and the low protein levels in the SEC-enriched samples indicated minimal contamination with freely circulating proteins. This is important, as the TBI markers of interest in this study are proteins, making it crucial to distinguish between freely circulating proteins and those derived from EVs. After detergent treatment, total and nerve-derived protein levels increased, suggesting that most of the measured protein originated from the EV cargo. The NTA analysis confirmed a narrow particle size distribution in the SEC-enriched samples of 70–130 nm, consistent with small EVs. Yet, NTA cannot differentiate EVs from non-vesicular particles with overlapping size profiles, such as lipoproteins (Comfort et al. 2018). hFCM was therefore used to further validate our findings, confirming the presence of EVs and their dissolution with detergent treatment. Sonication also reduced the CD9^+^CD63^+^CD81^+^ signal but did not increase total protein or biomarker concentration, likely due to protein aggregation from the sonication protocol. Subtracting detergent-treated hFCM signals from the total CD9^+^CD63^+^CD81^+^ signal calculated the EV-specific particle count, which showed minimal variation across donor pools. This contrasted with the significant variability observed in NTA measurements, likely reflecting contamination with non-EV particles, such as lipoproteins, from the use of non-fasting donor pools. While lipoprotein contamination is an inherent issue with SEC-enriched samples (Karimi et al. 2018), SEC offers the advantage of minimal contamination with freely circulating proteins, as shown in our validation. This is crucial, as most prior TBI studies have used precipitation-based methods (Mondello, et al. 2020; Guedes et al. 2022) that yield larger quantities of EVs (Veerman et al. 2021; Zhen, et al. 2022) with the cost of contamination with non-EV particles and freely circulating proteins (Yuana, et al. 2014; Sódar et al. 2016; Pang et al. 2020; Sjölin et al. 2022; Welton et al. 2015). Therefore, SEC provides a reliable platform for studies requiring precise EV biomarker measurements.

Using this method, we found increased EV-GFAP levels in TBI patients compared to non-TBI trauma patients. Few studies have examined nerve-derived biomarkers in circulating EV cargo in acute TBI. Mondello et al. (Mondello, et al. 2020) measured EV-GFAP, EV-NfL, EV-UCH-L1, and EV-Tau levels in circulating EVs during the first 5 days after TBI using a cohort of 21 moderate to severe TBI cases but without controls. In their study, EV-GFAP concentrations peaked early and declined over time after injury. Similarly, Guedes et al. (Guedes et al. 2022) studied levels of EV-GFAP, EV-UCH-L1, EV-NfL, and EV-Tau in 41 TBI patients and 73 trauma controls 6–12 h after injury, finding elevated EV-GFAP levels in TBI patients. Our results align with these studies, reinforcing EV-GFAP as a reliable TBI biomarker. Yet, EV-NfL, EV-T-Tau, and EV-UCH-L1 levels were unaltered in TBI patients in our study. This is in contrast to Guedes et al. (Guedes et al. 2022) who found elevated EV-NfL and EV-UCH-L1 but not EV-Tau in TBI patients, and may be attributed to contamination from freely circulating proteins in their precipitation-based method. The exact reasons for the discrepancy remain unclear, and further investigation is needed to determine whether the isolation method or other factors, such as injury severity or timing of sample collection, explain these differences.

EV-Tau has been more widely studied in cohorts of mild and repetitive TBI (Kawata et al. 2018; Goetzl et al. 2019; Kenney et al. 2018; Gill et al. 2018). Collectively, these studies reported elevated EV-Tau levels suggesting a biomarker potential in mild TBI. This highlights a potential biomarker-specific capability of EV cargo proteins, similar to findings with freely circulating proteins (Kaaber et al. 2024). Lastly, the association between EV-GFAP levels with two injury severity indicators aligns with the findings of Guedes et al. (Guedes et al. 2022), who demonstrated significant associations with increasing Head Abbreviated Injury Scores (HAIS). While HAIS is anatomically based and does not directly reflect brain injury (Foreman et al. 2007), the Marshall score and CT findings provide direct neuroimaging measures (Marshall et al. 1992), which substantiate an association between EV-GFAP levels and the extent of neurological injury.

In our study, the extent of the association between plasma-GFAP and EV-GFAP with TBI severity was comparable. This aligns with Guedes et al. (Guedes et al. 2022), who reported a strong correlation (*r* = 0.96) between EV- and plasma-GFAP levels, suggesting that both provide identical information on CNS injury severity. This suggests that EV-GFAP offers little additional biomarker information compared to the easier measurement of plasma-GFAP (Abdelhak et al. 2022) on the parameters tested in our study. However, EVs are considered highly stable structures that tolerate different preanalytical conditions, which is why they are likely to protect biomarkers in their cargo more efficiently compared with soluble plasma proteins. Moreover, EV-based biomarkers may reflect other aspects of TBI pathophysiology better than plasma biomarkers, although this remains unclear and calls for further investigation. At present, EV biomarker analysis remains technically complex and is not yet suited for routine clinical implementation.

Interestingly, despite the overall similarity between plasma- and EV-GFAP, their temporal patterns differed: plasma-GFAP remained elevated at all timepoints, while EV-GFAP was only elevated at admission and 15 h. The mechanisms underlying the release of EV-encapsulated versus freely circulating GFAP remain incompletely understood, but the observed difference may reflect involvement of distinct cellular processes. For instance, EV-mediated release likely involves an active and regulated pathway, including intracellular packaging and secretion, while freely circulating GFAP may be released through alternative mechanisms.

No association was observed between EV and plasma biomarkers with the tested outcomes. In this study, EV biomarkers were associated with acute measures of TBI severity (Fig. 3), but not with long-term outcomes (Supplementary Table S5), suggesting their primary utility may lie in early-phase assessment. This contrast earlier findings of an association between the tested plasma biomarkers and outcomes in this cohort (Kaaber et al. 2024) and is likely caused by the smaller sample size of the present study. Furthermore, Mondello et al. showed a potential prognostic role for EV-UCH-L1 trajectories in early mortality (Mondello, et al. 2020), though these patterns were observed in a small sample with high bias risk. Collectively, this suggests that EV biomarkers may hold potential on outcome parameters which require further investigation.

Some limitations should be considered. This study may have a relatively low statistical power, due to high CV of the analytical method combined with the modest cohort size. While this limits firm conclusions on insignificant findings, it also highlights the robustness of EV-GFAP findings. The EVs in our study were not CNS-specific and therefore, we cannot rule out a potential interference of EVs from peripheral nerves. However, the analyzed proteins are nerve-specific, and the bulk derive from CNS. As such, the observed protein changes would mainly derive from the CNS.

Another limitation derives from normalization of biomarker levels to initial plasma volume rather than to EV count. While some studies suggest TBI may increase circulating EV counts (Kuravi et al. 2017), our study focuses on the biomarker potential of the total cargo protein. Whether changes in biomarker levels occur due to altered EV counts or cargo load would have little impact for this purpose. Finally, case/control misclassification could affect results, as mild or subclinical TBIs may have been overlooked. Yet, our study’s use of neuroimaging and clinical assessments for control classification would largely exclude this risk.

## Conclusion

Our study demonstrates increased EV-GFAP levels in acute TBI patients. It uncovers an association between EV-GFAP levels and neuroimaging-derived severity indicators, but not with long-term clinical outcomes. Using thoroughly validated methods for EV isolation and protein cargo measurement, it uncovers comparable biomarker capabilities for plasma and EV-GFAP for the endpoints tested. This favors plasma over EV biomarker in moderate to severe TBI and calls for more detailed analysis to unravel if EV-GFAP may provide biomarker value on other parameters or endpoints.

## Supplementary Information

Below is the link to the electronic supplementary material.

Supplementary file1 (DOCX 849 KB)

## Data Availability

No datasets were generated or analysed during the current study.

## Abbreviations

AIS: Abbreviated Injury Scale
ATLS: Advanced trauma life support
CD: Cluster of differentiation
CNS: Central nervous system
CV%: Coefficient of variation percentage
EDTA: Ethylenediaminetetraacetic acid
EV: Extracellular vesicle
GFAP: Glial fibrillary acidic protein
GCS: Glasgow Coma Scale
GOSE: Glasgow Outcome Scale Extended
HAIS: Head Abbreviated Injury Scale
hFCM: High-resolution flow cytometry
ID: Identification
ISS: Injury Severity Score
LOD: Limit of detection
LLOQ: Lower limit of quantification
mTBI: Mild traumatic brain injury
NfL: Neurofilament light chain
NISS: New Injury Severity Score
NTA: Nanoparticle tracking analysis
PBS: Phosphate-buffered saline
RT: Room temperature
SEC: Size exclusion chromatography
Simoa: Single molecule array
T-Tau: Total Tau
TBI: Traumatic brain injury
UCH-L1: Ubiquitin carboxy-terminal hydrolase L1
