# Supplementary material for: Extracellular Vesicle Glial Fibrillary Acidic Protein as a Circulating Biomarker of Traumatic Brain Injury Severity
Source: J Mol Neurosci. 2025 May 23;75(2):69. doi: 10.1007/s12031-025-02360-5 (PMC12102119; doi:10.1007/s12031-025-02360-5)
Supplement: Supplementary file 1 — Supplementary file1 (DOCX 849 KB) [file 12031_2025_2360_MOESM1_ESM.docx]

Supplementary Material

Extracellular Vesicle Glial Fibrillary Acidic Protein as a Biomarker of Traumatic Brain Injury Severity

Ayad Babaee^a,b^, Thea Overgaard Wichmann^b,i^, Mikkel M Rasmussen^a,c^, Ole Brink^d^, Dorte Aalund Olsen^e^, Lars C. Borris^d^, Maj Lesbo^f^, Rikke Wehner Rasmussen^g^, Carlos Salomon^j,k^, Aase Handberg^g,h^, Maiken Mellergaard^g,h^, Claus V.B. Hviid^a,b,g,h^

Content

- **Table S1:** Technical Details of Plasma Citrate and Extracellular Vesicle Biomarker Analysis.
- **Table S2:** Data Availability for SEC-enriched samples and Plasma Citrate.
- **Figure S1:** Elution Profiles of 70 nm qEVoriginal Size Exclusion Columns.
- **Figure S2:** Biomarker concentration in plasma from patients with TBI and non-TBI.
- **Table S3**: Association between admission extracellular vesicle biomarker levels and TBI severity.
- **Table S4:** Association between admission plasma biomarker levels and TBI severity.
- **Table S5:** Association between admission extracellular vesicle biomarker levels, 1-year mortality and 6–12 months unfavorable outcome in TBI patients.
- **Table S6:** Association between admission plasma biomarker levels, 1-year mortality and 6–12 months unfavorable outcome in TBI patients.
- **Figure S3:** Gating strategy for determining EV concentrations.
- **Figure S4:** Raw flow cytometry data for the three SEC enriched Ev pools.
- **Figure S5:** Calibration of Apogee A60-Micro for EV Size Determination Using Rosetta Beads.
- **Table S7:** MIFlowCyt compliant item check list.
- **Table S8:** MIFlowCyt-EV compliant item check list.

**Table S1:** Technical Details of Plasma Citrate and Extracellular Vesicle Biomarker Analysis

|  | **GFAP** | **NfL** | **T-Tau** | **UCH-L1** |
| --- | --- | --- | --- | --- |
| **Kit inert specifications** | | | | |
| LLOQ, pg/mL | 9.38 | 0.500 | 0.125 | 9.38 |
| LOD, pg/mL | 1.18 | 0.096 | 0.037 | 2.43 |
| Assay range, pg/mL | 0 - 40000 | 0 - 2000 | 0 - 400 | 0 - 40000 |
| **Intermediate precision** | | | | |
| Plasma pool  CV%  Mean ± SD (pg/mL) | 8.8  233 ± 20.5 | 10.5  24 ± 2.5 | 21.8  1.1 ± 0.2 | 40.6  13.6 ± 5.5 |
| Q1  CV%  Mean ± SD (pg/mL) | 10.2  111 ± 11.3 | 11.3  6.3 ± 0.7 | 15.0  2.0 ± 0.3 | 11.7  134.1 ± 15.7 |
| Q2  CV%  Mean ± SD (pg/mL) | 5.2  9321 ± 481.1 | 9.6  443 ± 42.5 | 10.2  65.2 ± 6.6 | 10.8  10652.7 ± 1150.7 |

Table S1: Analytical performance specifications of the Simoa HD-X using the Neurology 4-plex assay B kit (Quanterix Corp, MA USA). Intermediate precision was evaluated using a plasma pool and two controls at low and high level. Abbreviations: T-Tau: Total-Tau, NfL: neurofilament light chain, GFAP: glial fibrillary acidic protein, UCH-L1: Ubiquitin carboxy-terminal hydrolase L1, LLOQ: Lower limit of quantification, LOD: Lower limit of detection, CV: Coefficient of Variation, Simoa: Single Molecule Array.

**Table S2:** Data Availability for SEC-enriched samples and Plasma Citrate.

**a**

| EV | EV-GFAP | EV-NfL | EV-Tau | EV-UCH-L1 |
| --- | --- | --- | --- | --- |
| <LOD (0) | 7 | 44 | 3 | 0 |
| <LOD (15) | 12 | 43 | 9 | 0 |
| <LOD (72) | 4 | 19 | 4 | 1 |
| Missing (0) | 3 | 3 | 3 | 3 |
| Missing (15) | 15 | 15 | 15 | 15 |
| Missing (72) | 48 | 48 | 48 | 48 |

**b**

| Plasma | GFAP | NFL | T-Tau | UCH-L1 |
| --- | --- | --- | --- | --- |
| <LOD (0) | 0 | 0 | 0 | 0 |
| <LOD (15) | 1 | 0 | 0 | 1 |
| <LOD (72) | 0 | 1 | 0 | 4 |
| Missing (0) | 3 | 3 | 3 | 3 |
| Missing (15) | 15 | 15 | 15 | 15 |
| Missing (72) | 48 | 48 | 48 | 48 |

Table S2: Counts of data under the limit of detection (LOD) or missing samples for the four biomarkers (T-T-Tau, NFL, GFAP, and UCH-L1) across three time points (on admission, 15 hours, and 72 hours). **(a)** shows data for biomarkers measured in extracellular vesicle isolates, and **(b)** shows data for biomarkers measured in plasma citrate. Abbreviations: NfL Neurofilament light, GFAP Glial fibrillary acidic protein, UCH-L1 Ubiquitin carboxy-terminal hydrolase L1

**Figure S1.** Elution Profiles of 70 nm qEVoriginal Size Exclusion Columns

**
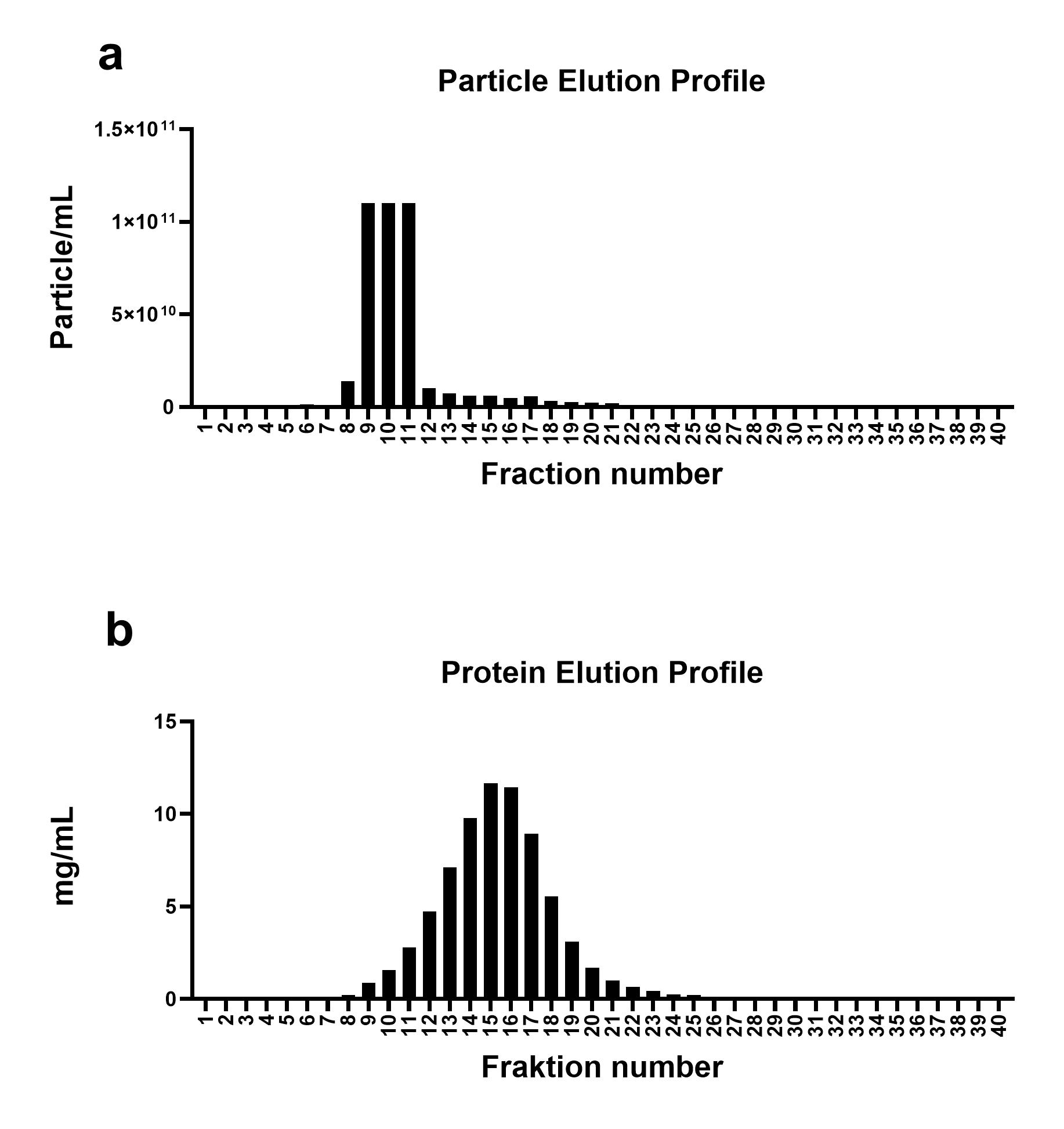
**

Figure 1: **(a)** Particle concentration in each fraction collected from 70 nm qEVoriginal size exclusion columns measured using NTA. A 500 µL pooled plasma sample was loaded onto the column, and 400 µL fractions were collected sequentially, up to a total of 40 fractions. **(b)** Total protein measurement of the same fractions analyzed using Nanodrop, with protein concentration (mg/mL) plotted against the fraction number. Abbreviations: NTA Nanoparticle Tracking Analysis.

**Figure S2:** Biomarker concentration in plasma from patients with TBI and non-TBI



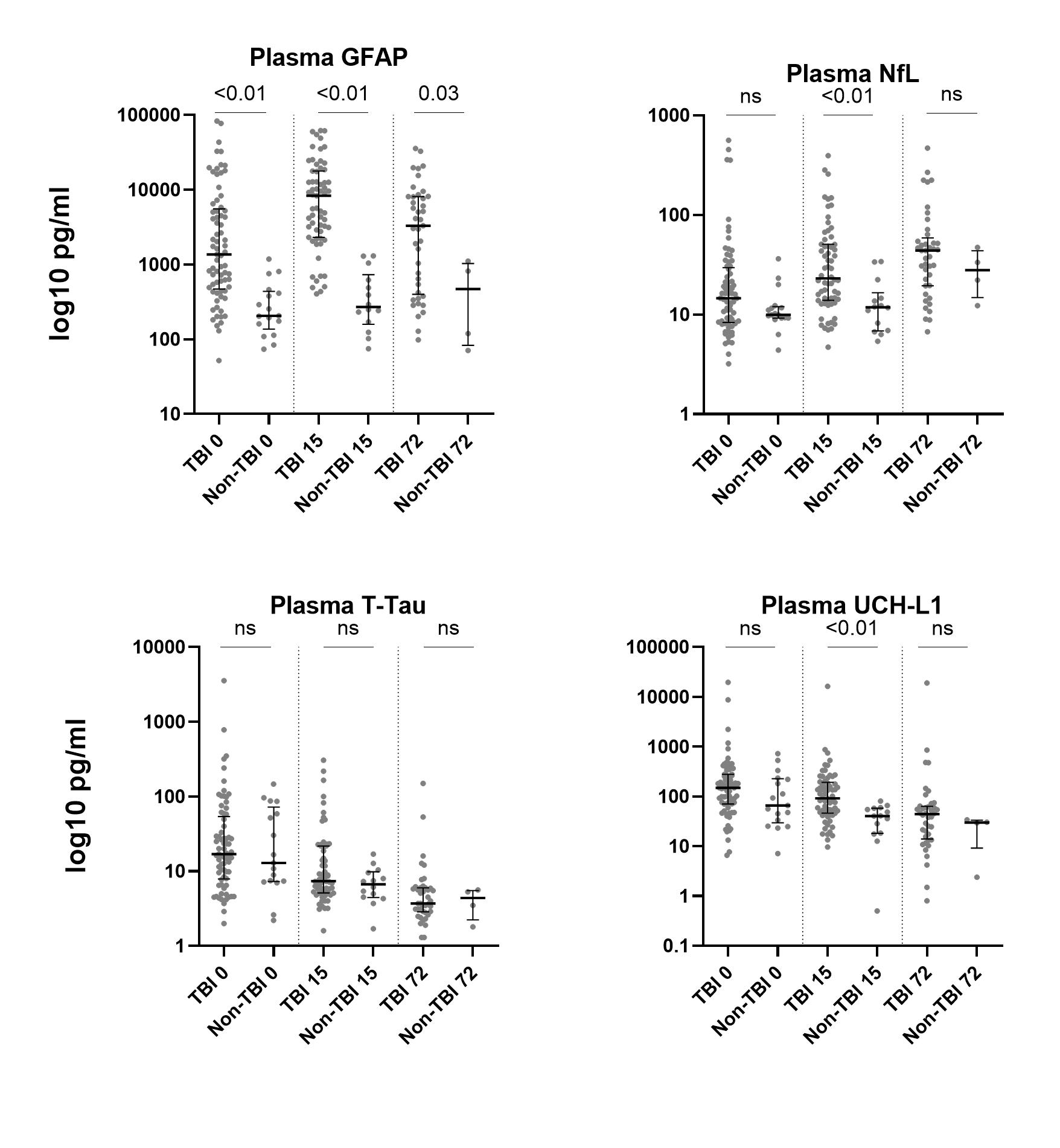


Figure S2: Biomarker concentration in plasma from patients with TBI and non-TBI on hospital admission (0h), and after 15- and 72 hours of hospital stay. Biomarker levels are presented on a logarithmic scale (log10 pg/mL). Values are presented as median with interquartile ranges (IQR). Abbreviations: TBI, traumatic brain injury, GFAP glial fibrillary acidic protein, NfL neurofilament light chain, T-Tau total Tau, UCH-L1 ubiquitin carboxy-terminal hydrolase-L1, ns not significant

| **Table S3:** Association between admission extracellular vesicle biomarker levels and TBI severity | | | | | | | | |
| --- | --- | --- | --- | --- | --- | --- | --- | --- |
|  | EV-GFAP | | EV-NfL | | EV-Tau | | EV-UCH-L1 | |
|  | Crude | Adjusted | Crude | Adjusted | Crude | Adjusted | Crude | Adjusted |
| GCS | 0.57  (0.33 -0.99)  p = 0.04 | 0.64  (0.35 - 1.17)  p = 0.15 | 0.68  (0.35 - 1.30)  p = 0.24 | 0.54  (0.26 - 1.10)  p = 0.08 | 0.79  (0.55-1.14)  p = 0.22 | 0.77  (0.52 - 1.15)  p = 0.21 | 0.86  (0.64 - 1.16)  p = 0.33 | 0.78  (0.56 - 1.10)  p = 0.13 |
| CT lesion | 3.1  (1.30 - 7.34)  p = 0.01 | 2.85  (1.18 - 6.91)  p = 0.02 | 1.52  (0.51 - 4.49)  p = 0.44 | 1.77  (0.58 - 5.30)  p = 0.3 | 1.11  (0.61 - 2.02)  p = 0.73 | 1.11  (0.60 - 2.01)  p = 0.73 | 0.89  (0.54 - 1.47)  p = 0.65 | 0.95  (0.57 - 1.57)  p = 0.84 |
| Marshall score | 2.19  (1.29 - 3.73)  p < 0.01 | 2.11  (1.19 - 3.77)  p = 0.01 | 1.12  (0.58 - 2.16)  p = 0.72 | 1.39  (0.69 - 2.82)  p = 0.34 | 1.10  (0.74 - 1.58)  p = 0.65 | 1.10  (0.73 - 1.63)  p = 0.65 | 0.90  (0.66 - 1.22)  p = 0.51 | 0.99  (0.72 - 1.38)  p = 0.97 |

Table S3: Multiple linear regression analyses of the association between admission extracellular vesicle biomarker levels and TBI severity. Categorized admission GCS (14–15, 9–13, 3–8), presence of lesion on head CT (yes/no), and Marshall Classification score (I, II–IV, V–VI). Adjustments for age, sex, and NISS. The coefficients are presented with 95% confidence intervals and corresponding p-values. TBI traumatic brain injury, GCS Glasgow Coma Scale, CT computed tomography, NISS new injury severity score, NfL neurofilament light, GFAP glial fibrillary acidic protein, UCH-L1 ubiquitin carboxy-terminal hydrolase L1.

| **Table S4:** Association between admission plasma biomarker levels and TBI severity | | | | | | | | |
| --- | --- | --- | --- | --- | --- | --- | --- | --- |
|  | GFAP | | NfL | | T-Tau | | UCH-L1 | |
|  | Crude | Adjusted | Crude | Adjusted | Crude | Adjusted | Crude | Adjusted |
| GCS | 0.40  (0.24 - 0.65)  p < 0.001 | 0.61  (0.37 - 0.98)  p = 0.06 | 0.54  0.40-0.73)  p < 0.001 | 0.72  (0.53 - 0.96)  p = 0.02 | 0.79  (0.51 - 1.23)  p = 0.30 | 1.04  (0.7 - 1.53)  p = 0.85 | 0.58  (0.39 - 0.88)  p = 0.01 | 0.73  (0.47 - 1.11)  p = 0.14 |
| CT lesion | 4.64  (2.11 - 10.22)  P < 0.001 | 3.4  (1.72 - 6.70)  p < 0.001 | 1.57  (0.93 - 2.66)  p = 0.08 | 1.3  (0.84 - 2.02)  p = 0.22 | 1.11  (0.55 - 2.26)  p = 0.75 | 0.87  (0.5 - 1.55)  p = 0.65 | 0.65  (0.33 - 1.27)  p = 0.21 | 0.52  (0.28 - 0.95)  p = 0.03 |
| Marshall score | 2.88  (1.78 - 4.65)  p < 0.001 | 2.1  (1.33 - 3.31)  p < 0.01 | 1.4  (1.01 - 1.93)  p = 0.04 | 1.17  (0.88 - 1.57)  p = 0.26 | 1.19  (0.77 - 1.85)  p = 0.41 | 0.86  (0.59 - 1.25)  p = 0.43 | 0.9  (0.59 - 1.39)  p = 0.66 | 0.64  (0.43 - 0.95)  p = 0.03 |

Table S4: Multiple linear regression analyses of the association between admission plasma citrate biomarker levels and TBI severity. Categorized admission GCS (14–15, 9–13, 3–8), presence of lesion on head CT (yes/no), and Marshall Classification score (I, II–IV, V–VI). Adjustments for age, sex, and NISS. The coefficients are presented with 95% confidence intervals and corresponding p-values. TBI traumatic brain injury, GCS Glasgow Coma Scale, CT computed tomography, NISS new injury severity score, NfL neurofilament light, GFAP glial fibrillary acidic protein, UCH-L1 ubiquitin carboxy-terminal hydrolase L1.

**Table S5** Association between admission extracellular vesicle biomarker levels, 1-year mortality and 6–12 months unfavorable outcome in TBI patients.

| One year mortality | | | | | | | | | | |  |
| --- | --- | --- | --- | --- | --- | --- | --- | --- | --- | --- | --- |
| Biomarker | | Crude | | Model 1 | Model 2 | | | Model 3 | Model 4 | |  |
| EV-GFAP (per 100 pg/mL) | 0.99  (0.83 - 1.19)  p = 0.98 | | 1.11  (0.82 - 1.48)  p = 0.48 | | 1.11  (0.86 - 1.43)  p = 0.41 | | 1.04  (0.76 - 1.44)  p = 0.79 | | 1.11  (0.86 - 1.43)  p = 0.42 | |  |
| EV-NfL (per 1 pg/mL) | 0.86  (0.54 - 1.38)  p = 0.52 | | 0.92  (0.57 - 1.47)  p = 0.72 | | 0.88  (0.49 - 1.57)  p = 0.67 | | 0.62  (0.15 - 2.61)  p = 0.52 | | 0.77  (0.22 - 2.65)  p = 0.67 | |  |
| EV-Tau (per 1 pg/mL) | 0.85  (0.49 - 1.45)  p = 0.55 | | 0.96  (0.5 - 1.85)  p = 0.92 | | 0.93  (0.49 - 1.79)  p = 0.84 | | 0.84  (0.33 - 2.14)  p = 0.72 | | 0.8  (0.27 - 2.33)  p = 0.69 | |  |
| EV-UCH-L1 (per 100 pg/mL) | | 0.78  (0.36 - 1.70)  p = 0.55 | | 0.92  (0.45 - 1.86)  p = 0.81 | | 0.87  (0.36 - 2.07)  p = 0.75 | | 0.78  (0.19 - 3.19)  p = 0.73 | | 0.83  (0.29 - 2.34)  p = 0.73 | |
| 6–12 months unfavorable outcome | | | | | | | | | | |  |
| EV-GFAP (per 100 pg/mL) | 1.13  (0.91 - 1.41)  p = 0.26 | | 1.19  (0.94 - 1.51)  p = 0.15 | | 1.26  (0.97 - 1.63)  p = 0.08 | | 1.15  (0.90 - 1.46)  p = 0.25 | | 1.24  (0.95 - 1.59)  p = 0.11 | |  |
| EV-NfL (per 1 pg/mL) | 1.01  (0.92 - 1.12)  p = 0.79 | | 1.07  (0.95 - 1.20)  p = 0.24 | | 1.07  (0.96 - 1.21)  p = 0.19 | | 1.05  (0.94 - 1.17)  p = 0.36 | | 1.06  (0.95 - 1.19)  p = 0.29 | |  |
| EV-Tau (per 1 pg/mL) | 1.02  (0.79 - 1.32)  p = 0.84 | | 1.5  (0.87 - 1.53)  p = 0.34 | | 1.19  (0.88 - 1.61)  p = 0.24 | | 1.1  (0.83 - 1.46)  p = 0.49 | | 1.15  (0.84 - 0.58)  p = 0.36 | |  |
| EV-UCH-L1(per 100 pg/mL) | 1.10  (0.87 - 1.39)  p = 0.41 | | 1.22  (0.93 - 1.59)  p = 0.15 | | 1.28  (0.97 - 1.68)  p = 0.07 | | 1.19  (0.93 - 1.54)  p = 0.16 | | 1.28  (0.97 - 1.68)  p = 0.08 | |  |

**Table S5** Results of logistic regression analyses of the association between admission levels of extracellular vesicle biomarkers and 1-year mortality and 6–12 months unfavorable outcome (GOSE ≤ 4). Odds ratios (OR) with 95% confidence intervals presented. Model adjustments: Model 1: NISS, age; Model 2: GCS on admission, age; Model 3: Marshall Classification Score, age; Model 4: Pupil reflex, admission GCS, and age. Abbreviations: NISS new injury severity score, GCS Glasgow coma scale, GFAP fibrillary acidic protein, NfL neurofilament light, T-Tau Total Tau, UCH-L1 ubiquitin carboxyl-terminal hydrolase L1.

**Table S6**: Association between admission levels of plasma citrate biomarkers and 1-year mortality and 6–12 months unfavorable outcome in TBI patients.

| One year mortality | | | | | |
| --- | --- | --- | --- | --- | --- |
| Biomarker | Crude | Model 1 | Model 2 | Model 3 | Model 4 |
| GFAP (per 10.000 pg/mL) | 1.52  (1.05 - 2.19)  p = 0.03 | 1.62  (0.90 - 2.90)  p = 0.11 | 1.71  (1.00 - 2.92)  p = 0.05 | 1.5  (0.83 - 2.69)  p = 0.17 | 1.60  (0.95 - 2.67)  p = 0.07 |
| NfL (per 100 pg/mL) | 1.58  (0.96 - 2.62)  p = 0.07 | 1.44  (0.81 - 2.53)  p = 0.21 | 1.61  (0.91 - 2.83)  p = 0.09 | 1.55  (0.87 - 2.77)  p = 0.13 | 1.55  (0.89 - 2.70)  p = 0.12 |
| T-Tau (per 1000 pg/mL) | 1.01  (0.21 - 4.85)  p = 0.99 | 1.06  (0.13 - 8.30)  p = 0.95 | 1.60  (0.28 - 8.77)  p = 0.59 | 1.25  (0.23 - 1.25)  p = 0.81 | 1.04  (0.15 - 6.92)  p = 0.96 |
| UCH-L1 (per 100 pg/mL) | 0.99  (0.95 - 1.03)  p = 0.78 | 0.99  (0.95 - 1.04)  p = 0.84 | 0.99  (0.96 - 1.03)  p = 0.97 | 0.99  (0.96 - 1.04)  p = 0.99 | 0.99  (0.95 - 1.04)  p = 0.84 |
| 6–12 months unfavorable outcome | | | | | |
| GFAP (per 10.000 pg/mL) | 2.04  (1.16 - 3.59)  p = 0.01 | 1.74  (0.99 - 3.14)  p = 0.07 | 1.74  (0.96 - 3.15)  p = 0.07 | 1.66  (0.93 - 2.99)  p = 0.09 | 1.73  (0.87 - 3.45)  p = 0.12 |
| NfL (per 100 pg/mL) | 30.98  (1.29 - 742.86)  p = 0.03 | 8.77  (0.34 - 228.47)  p = 0.19 | 20.36  (0.45 - 914.40)  p = 0.12 | 13.74  (0.45 - 416.80)  p = 0.13 | 14.66  (0.22 - 976.00)  p = 0.21 |
| T-Tau (per 100 pg/mL) | 1.21  (0.82 - 1.80)  p = 0.34 | 1.12  (0.85 - 1.49)  p = 0.42 | 1.16  (0.79 - 1.71)  p = 0.43 | 1.15  (0.81 - 1.65)  p = 0.42 | 0.56  (0.11 - 2.86)  p = 0.49 |
| UCH-L1 (per 100 pg/mL) | 1.08  (0.94 - 1.24)  p = 0.28 | 1.04  (0.95 - 1.14)  p = 0.38 | 1.05  (0.94 - 1.17)  p = 0.36 | 1.06  (0.92 - 1.23)  p = 0.41 | 1.03  (0.97 - 1.09)  p = 0.31 |

Table S6: Results of logistic regression analyses of the association between admission levels of plasma citrate biomarkers and 1-year mortality and 6–12 months unfavorable outcome. Odds ratios (OR) with 95% confidence intervals presented. Model adjustments: Model 1: NISS, age; Model 2: GCS on admission, age; Model 3: Marshall Classification Score, age; Model 4: Pupil reflex, admission GCS, and age. NISS new injury severity score, GCS Glasgow coma scale, NfL neurofilament light, GFAP fibrillary acidic protein, UCH-L1 ubiquitin carboxyl-terminal hydrolase L1.

**Figure S3**

**
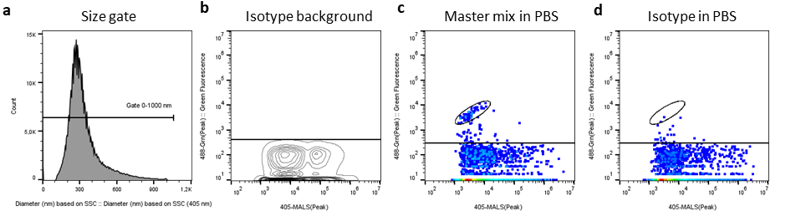
**

**Figure S3.** Gating strategy for determining EV concentrations. Firstly, samples were calibrated using the Rosetta Calibration software v2.05 (Exometry, Amsterdam, The Netherlands) and a 0-1000 nm gate was applied to all samples (a). Positive events were defined according to the isotype background set using a contour plot at a 2% level (b). Antibody aggregates were gated on antibody mixes in solution analyzed in PBS (c and d). This gate was transferred to all samples to remove potential false-positive events (derived from antibody aggregates) from the final EV concentrations (Fig S4). Abbreviations: PBS phosphate buffered saline.

**Figure S4**

**
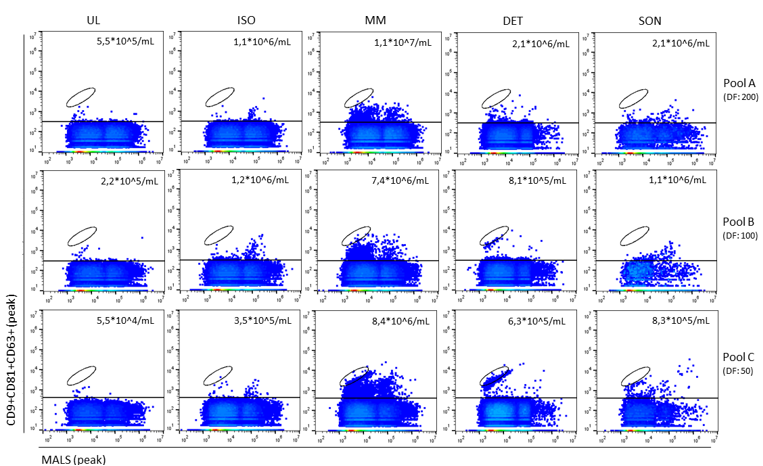
**

**Figure S4.** Raw flow cytometry data for the three SEC enriched Ev pools. Final measured EV concentration (or background measurements) in each sample in upper right corners. UL Unlabelled, ISO isotype, MM, master mix, DET detergent, SON sonication.

**Figure S5**

**A B**

**
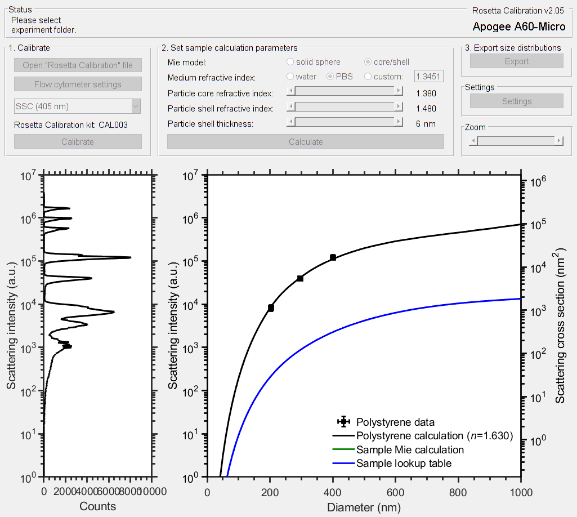

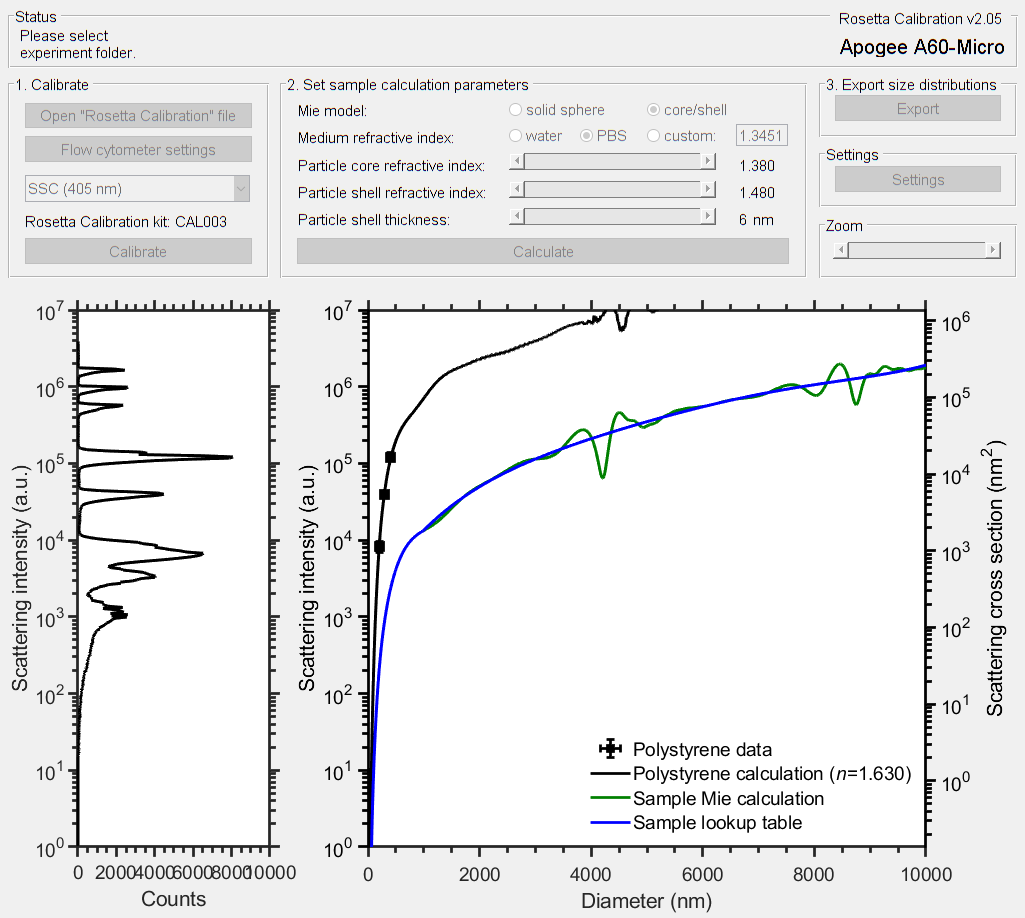
**

**Figure S5.**

Rosetta beads were run at our Apogee A60-Micro at same settings as the sample runs. The obtained fcs-beads file was loaded in the Rosetta calibration software v.2.05, settings selected for Apogee A60-Micro, SSC (405nm), and sample calculation parameters selected as recommended by the manufacturer for EV in PBS. A) Based on the optical configuration of the FCM, Mie calculations and optical properties and known size of the beads, a graph relating scattering intensity to size is obtained. B) considering the optical properties of EVs in PBS, a graph relating scatter intensity to size of EV is obtained. This calculation is exported to fcs files of the samples, and an extra size(nm) dimension are added to the files. Abbreviations: PBS phosphate buffered saline, FCM flow cytometry, SSC side scatter, FCS flow cytometry standard, A.U arbitrary unit.

**Table S7: MIFlowCyt compliant item check list.**

| **Requirement** | **Please Include Requested Information** |
| --- | --- |
| 1.1. Purpose | Validate EV concentrations in SEC enriched samples |
| 1.2. Keywords | Extracellular vesicles; Size Exclusion Chromatography; High resolution flow cytometry; Intra-vesicular content; NTA; |
| 1.3. Experiment variables | SEC purification; staining of EVs; Lysis/sonication of EVs; |
| 1.4. Organization name and address | Department of Clinical Biochemistry, Aalborg University Hospital, North Denmark Region, Hobrovej 18-22, DK-9000 Aalborg, Denmark.  Department of Clinical Medicine, Aalborg University, Sdr. Skovvej 15, DK-9000 Aalborg, Denmark.  Department of Clinical Biochemistry, Aarhus University Hospital, Palle Juul-Jensens Blvd. 99, DK-8200 Aarhus, Denmark. |
| 1.5. Primary contact name and email address | Claus Vinter Bødker Hviid ([claus.hviid@rn.dk](mailto:claus.hviid@rn.dk)).  Maiken Mellergaard ([maiken.pedersen@rn.dk](mailto:maiken.pedersen@rn.dk)). |
| 1.6. Date or time period of experiment | February-May 2024 |
| 1.7. Conclusions | The use of SEC demonstrated consistency in particle size distribution, confirmed the presence of EV markers (CD9, CD63, CD81), and enabled reliable protein cargo measurement, establishing SEC as a robust EV isolation method for biomarker studies in trauma-related research. |
| 1.8. Quality control measures | **Daily system stability controls:**  Light scatter: Apogee Mix bead mixture (Apogee Flow Systems, Cat. no. 1527, Lot. no. CAL0172).  Fluorescence: Ultra Rainbow Calibration particles (Spherotech, Lake Forest, IL, USA, Cat. no. RCP-30-5, Lot. no. AP04).  Background stability: Buffer alone control (PBS).  **Sample-specific controls:**  Unstained buffer (PBS)  Isotype mix in PBS (Ab aggregate control)  Master mix in PBS (Ab aggregate control)  Unstained samples  Stained samples – isotype mix  Stained samples – master mix  Detergent lysis of Stained samples – master mix  Sonication of Stained samples – master mix  **Titrations:**  Single isotype/antibody titration  Sample serial dilution. |
| 2.1.1.1. (2.1.2.1., 2.1.3.1.) Sample description | SEC enriched samples from human plasma (described in materials and methods section) |
| 2.1.1.2. Biological sample source description | Human plasma (described in materials and methods section) |
| 2.1.1.3. Biological sample source organism description |  |
| 2.1.2.2. Environmental sample location |  |
| 2.3. Sample treatment description | **Staining and preparation for analysis:**  SEC enriched samples were thawed at room temperature before staining.  Prior to staining antibody and isotype mixes were prepared in PBS and filtered through 0,45 µm centrifugation filters (Millipore, Cat. UCF30HVNB) at 12000xg for 10 min at 20°C to reduce antibody aggregates.  Staining was performed by mixing 90 µL of SEC enriched sample and 50 µL antibody or isotype mixes or PBS (for unstained control) prior to staining at room temperature in the dark for 2 hours.  After incubation, samples were diluted individually according to dilution factor determined by titrating unstained sample (dilution factor between 50-200 (see figure S2) to obtain a flowrate of 2000-4000 events/s.  Acquisition of each sample was followed by a two-step cleaning procedure with PBS and two flushing cycles (to keep the background low). |
| 2.4. Fluorescence reagent(s) description | **Antibodies:**  FITC-conjugated mouse monoclonal anti-CD9 (Clone HI9A; Biolegend, Cat. no. 312104, Lot. no. B359149, Stock concentration: 100 *μ*g/ml).  FITC-conjugated mouse monoclonal anti-CD81 (Clone 5A6; Biolegend, Cat. no. 349504, Lot. no. B383514; Stock concentration: 200 *μ*g/ml).  FITC-conjugated mouse monoclonal anti-CD63 (Clone H5C6; Biolegend, Cat. no. 353006, Lot. no. B262101; Stock concentration: 200 *μ*g/ml)  **Isotype control:**  FITC-conjugated mouse IgG1*κ* (Clone MOPC-21; Biolegend, Cat. no. 400110, Lot. no. B283622; Stock concentration: 200 *μ*g/ml).  **Titrations:**  Titration of all antibodies and isotypes were performed to define optimal concentration ensuring optimal concentration without excess to enhance risk of unspecific binding and antibody aggregation. Final concentrations were used for mixes as described:  Antibody mixes were prepared by adding FITC-CD9 (2.5 µL/sample), FITC-CD81 (3 µL/sample), FITC-CD63 (2.5 µL/sample) in 42 µL PBS/sample (in total = 50 µL antibody mix/sample).  Isotype control mixes (background controls) were prepared by adding FITC-IgG1κ isotype control (6.75 µL/sample) in 43.25 µL PBS/sample (in total = 50 µL isotype mix/sample). |
| 3.1. Instrument manufacturer | Apogee Flow Systems, Hemel Hempstead, UK |
| 3.2. Instrument model | A60 Micro-PLUS |
| 3.3. Instrument configuration and settings | Instrument configuration and settings:  Lasers:   - 405nm: 140mW - 488nm: 100mW   PMTs:   - SALS: 400V - MALS: 380V, threshold: 27 - LALS: 400V - 488Green: 400V, Filter: 530/40BP   Sample flow rate: 3.01 μl/min, aspirating 120 µL with acquisition time set to 180s. |
| 4.1. List-mode data files | Flow cytometry data can be acquired via contact to Claus Vinter Bødker Hviid ([claus.hviid@rn.dk](mailto:claus.hviid@rn.dk)) or Maiken Mellergaard ([maiken.pedersen@rn.dk](mailto:maiken.pedersen@rn.dk)). |
| 4.2. Compensation description | Compensation was not performed due to use of a single channel. |
| 4.3. Data transformation details | Data was not transformed. |
| 4.4.1. Gate description | Gates were set according to isotype controls (see figure S1 for example). Antibody-positive events were defined as having a fluorescent signal higher than the lower boundary of the gate (see figure S1 and S2 for example). |
| 4.4.2. Gate statistics | Concentrations were calculated as number of events divided by sample volume and multiplied by dilution factor. |
| 4.4.3. Gate boundaries | Gate boundaries were defined according to Rosetta beads gating on size: 0-1000 nm (see figure S1). |

**Table S8: MIFlowCyt-EV compliant item check list.**

| **Framework Criteria** | **What to report** | **Please complete each criterion** |
| --- | --- | --- |
| 1.1 Preanalytical variables conforming to MISEV guidelines. | Preanalytical variables relating to EV sample including source, collection, isolation, storage, and any others relevant and available in the performed study. | Blood samples were obtained either from an arterial line or by venous puncture, and collected into citrated, heparin or EDTA tubes (BD Vacutainer®, Becton, Dickinson and Company, Franklin Lakes, NJ, USA). Samples were transported to the laboratory at room temperature and processed within one hour. |
| 1.2 Experimental design according to MIFlowCyt guidelines. | EV-FC manuscripts should provide a brief description of the experimental aim, keywords, and variables for the performed FC experiment(s) using MIFlowCyt checklist criteria: 1.1, 1.2, and 1.3, respectively | **Aim:**  Validate size exclusion chromatography (SEC) as a reliable method for EV isolation.  Investigate associations between EV biomarkers and TBI severity.  Explore the relationship between EV biomarkers and long-term clinical outcomes.  **Keywords:** TBI, GFAP, Extracellular vesicles; Size Exclusion Chromatography; High resolution flow cytometry; Intra-vesicular content; NTA.  **Variables:** SEC purification; staining of EVs; Lysis/sonication of EVs. |
| 2.1 Sample staining details | State any steps relating to the staining of samples. Along with the method used for staining, provide relevant reagent descriptions as listed in MIFlowCyt guidelines (Section 2.4 Fluorescence Reagent(s) Descriptions). | **Reagents:**  Antibodies:  FITC-conjugated mouse monoclonal anti-CD9 (Clone HI9A; Biolegend, Cat. no. 312104, Lot. no. B359149, Stock concentration: 100 *μ*g/ml).  FITC-conjugated mouse monoclonal anti-CD81 (Clone 5A6; Biolegend, Cat. no. 349504, Lot. no. B383514; Stock concentration: 200 *μ*g/ml).  FITC-conjugated mouse monoclonal anti-CD63 (Clone H5C6; Biolegend, Cat. no. 353006, Lot. no. B262101; Stock concentration: 200 *μ*g/ml)  Isotype control:  FITC-conjugated mouse IgG1*κ* (Clone MOPC-21; Biolegend, Cat. no. 400110, Lot. no. B283622; Stock concentration: 200 *μ*g/ml).  **Staining and preparation for analysis:**  Prior to labelling, SEC enriched samples were thawed at room temperature.  Prior to staining antibody and isotype mixes were prepared in PBS and filtered through 0,45 µm centrifugation filters (Millipore, Cat. UCF30HVNB) at 12000xg for 10 min at 4°C to reduce antibody aggregates.  Staining was performed by mixing 90 µL of SEC enriched sample and 50 µL antibody or isotype mixes or PBS (for unstained control) prior to staining at room temperature in the dark for 2 hours. After incubation, samples were diluted individually according to dilution factor determined by titrating unstained sample (dilution factor between 50-200 (see figure S2) to obtain a flowrate of 2000-4000 events/s. |
| 2.2 Sample washing details | State any steps relating to the washing of samples. | No sample washing was performed as all samples were stained directly in PBS-antibody mixes and only further diluted in PBS prior to flow cytometry analysis. |
| 2.3 Sample dilution details | All methods and steps relating to sample dilution. | All samples were diluted 1:1.5 for staining (in master mix, isotype mix, or PBS; 90 µL of SEC enriched sample was mixed with 50 µL master mix, isotype mix, or PBS). After incubation samples were diluted individually to obtain similar flowrate (2000-4000 events/sec) when analyzed by flow cytometry. Dilution factors were determined by running serial dilutions of unstained samples in PBS to reach the optimal flowrate for each sample. Each sample was then diluted accordingly in PBS. |
| 3.1 Buffer alone controls. | State whether a buffer-only control was analyzed at the same settings and during the same experiment as the samples of interest. If utilized it is recommended that all samples be recorded for a consistent set period of time e.g. 5 minutes, rather than stopping analysis at a set recorded event count e.g. 100,000 events. This allows comparisons of total particle counts between controls and samples. | Buffer alone (PBS) controls were included every day at start up as well as between each sample after cleaning solution to monitor and ensure stable (and low, defined as < 10 events/sec) background of the instrument. PBS between samples were analyzed according to the same settings as the samples (described table S1, 3.3). |
| 3.2 Buffer with reagent controls. | State whether a buffer with reagent control was analyzed at the same settings, same concentrations, and during the same experiment as the samples of interest. If used state what the results were. | Buffer (PBS) with master mixes or isotype mixes (Ab-aggregate controls) were included to access and define background deriving from antibody aggregates. Ab-aggregate controls were analyzed according to the same settings as the samples (described table S1, 3.3). |
| 3.3 Unstained controls. | State whether unstained control samples were analyzed at the same settings and during the same experiment as stained samples. If used, state what the results were, preferably in standard units. | Unstained sample controls were included for each sample to access and define background autofluorescence. Unstained controls were analyzed according to the same settings as the samples (described table S1, 3.3). |
| 3.4 Isotype controls. | The use of isotype controls is applicable to immunofluorescence labelling only. State whether isotype controls were analyzed at the same settings and during the same experiment as stained samples. If utilized, state which antibody they are matched to, the concentration used, and what the results were (Section 4.2, 4.3, 4.4). Due to conjugation differences between manufacturers if should be stated if the isotype controls are from the same manufacturer as the matched antibodies. | Isotype controls were included for each sample to access and define background and possible unspecific antibody binding. Isotype controls were analyzed according to the same settings as the samples (described table S1, 3.3). Isotype controls were purchased from the same manufacturer as the matched antibodies (see details in section.  2.1) |
| 3.5 Single-stained controls. | State whether single-stained controls were included. If used state whether the single-stained controls were recorded using the same settings, dilutions, and during the same experiment as stained samples and state what the results were, preferably in standard units (Section 4.2, 4.3, 4.4). | Not applicable. |
| 3.6 Procedural controls. | State whether procedural controls were included. If used, state the procedure and if the procedural controls were acquired at the same settings and during the same experiment as stained samples. | No procedural controls were included. |
| 3.7 Serial dilutions. | State whether serial dilutions were performed on samples and note the dilution range and manner of testing. The fluorescence and/or scatter signal intensity would ideally be reported in standard units (see Section 4.3, 4.4) but arbitrary units can also be used. This data is best reported by plotting the recorded number events/concentration over a set period of time at different sample dilution. The median fluorescence intensity at each of the dilutions should also ideally be plotted on the same or a separate plot. | Serial dilutions of stained samples were not performed. |
| 3.8. Detergent treated EV-samples | State whether samples were detergent treated to assess lability. If utilized, state what detergent was used, the end concentration of the detergent, and what the results were of the lysis. | Detergent lysis controls or sonication were included for all (antibody master mix) stained samples. Detergent lysis was done by: incubating stained samples in a 1 % (final concentration) Triton X-100 (Merck, Cat. 93443-100mL) for at least 30 minutes at room temperature in the dark. Sonication was done with a Bioruptor UCD-200 sonication device (Diagenode, Liège, Belgium). SEC-enriched samples were placed in a cooling bath to maintain a consistent temperature and prevent overheating during the sonication process. The sonication was performed in cycles. Sonication for 30 seconds (ON) followed by a 15-second rest period (OFF) constituted one cycle. This process was repeated for a total of 6 cycles.  Detergent lysis/sonication controls were analyzed according to the same settings as the samples (described table S1, 3.3). |
| 4.1 Trigger Channel(s) and Threshold(s). | The trigger channel(s) and threshold(s) used for event detection. Preferably, the fluorescence calibration (Section 4.3) and/or scatter calibration (Section 4.4) should be used in order to report the trigger channel(s) and threshold(s) in standardized units. | A triggering threshold was set on medium-angle light scatter (MALS) to a value of 27. This setting allowed the collection of less than 100 events/second in unstained PBS. |
| 4.2 Flow Rate / Volumetric quantification. | State if the flow rate was quantified/validated and if so, report the result and how they were obtained. | Optimal flowrate was based on optimization for another study (manuscript in preparation). Optimal flowrate was found to be 2000-4000 events/sec. |
| 4.3 Fluorescence Calibration. | State whether fluorescence calibration was implemented, and if so, report the materials and methods used, catalogue numbers, lot numbers, and supplied reference units for the standards. Fluorescence parameters may be reported in standardized units of MESF, ERF, or ABC beads. The type of regression used, and the resulting scatter plot of arbitrary data vs standard data for the reference particles should be supplied. | Fluorescence calibration was not performed. |
| 4.4 Light Scatter Calibration. | State whether and how light scatter calibration was implemented. Light scatter parameters may be reported in standardized units of nm2, along with information required to reproduce the model. | Rosetta beads (CAL003, Rosetta Calibration software ver. 2.05) according to settings for Apogee A60 Micro, SSC(405nm), Mie model Core/Shell, PBS, settings as preset in Rosetta Cal: particle core refractive index: 1.380, particle shell refractive index: 1.480, particle shell thickness 6nm (Fig S3). Rosetta beads were run at same settings as samples (described table S1, 3.3). Obtained scatter to diameter relations were added to fcs files. |
| 5.1 EV diameter/surface area/volume approximation. | State whether and how EV diameter, surface area, and/or volume has been calculated using FC measurements. | Rosetta beads (CAL003, Rosetta Calibration software ver. 2.05) according to settings for Apogee A60 Micro, SSC(405nm), Mie model Core/Shell, PBS, settings as preset in Rosetta Cal: particle core refractive index: 1.380, particle shell refractive index: 1.480, particle shell thickness 6nm (Fig S3). Rosetta beads were run at same settings as samples (described table S1, 3.3). Obtained scatter to diameter relations were added to fcs files. |
| 5.2 EV refractive index approximation. | State whether the EV refractive index has been approximated and how this was done. | EV refractive index was not approximated in this study. |
| 5.3 EV epitope number approximation. | State whether EV epitope number has been approximated, and if so, how it was approximated. | Not relevant. |
| 6.1 Completion of MIFlowCyt checklist. | Complete MIFlowCyt checklist criteria 1 to 4 using the MIFlowCyt guidelines. | MIFlowCyt checklist has been completed and is provided separately (See supplementary table S1). |
| 6.2 Calibrated channel detection range | If fluorescence or scatter calibration has been carried out, authors should state whether the upper and lower limits of a calibrated detection channel were calculated in standardized units. This can be done by converting the arbitrary unit scale to a calibrated scaled, as discussed in Section 4.3 and 4.4, and providing the highest unit on this scale and the lowest detectable unit above the unstained population. The lowest unit at which a population is deemed ‘positive’ can be determined a variety of ways, including reporting the 99th percentile measurement unit of the unstained population for fluorescence. The chosen method for determining at what unit an event was deemed positive should be clearly outlined. | Fluorescence calibration was not performed. |
| 6.3 EV number/concentration. | State whether EV number/concentration has been reported. If calculated, it is preferable to report EV number/concentration in a standardized manner, stating the number/concentration between a set detection range. | EV concentration was reported as EVs/mL, calculated by the equation:  EVs/mL = ((CD9^+^CD81^+^CD63^+^-positive EVs) / (volume of the run sample(pl))) * sample dilution factor * 1000000 |
| 6.4 EV brightness. | When applicable, state the method by which the brightness of EVs is reported in standardized units of scatter and/or fluorescence. | Not applicable. |
| 7.1. Sharing of data to a public repository. | Provide a link to the experimental data in a public data repository. | Contact Claus Vinter Bødker Hviid ([claus.hviid@rn.dk](mailto:claus.hviid@rn.dk)) or Maiken Mellergaard ([maiken.pedersen@rn.dk](mailto:aaha@rn.dk)). |
